# Nano-graphene oxide improved the antibacterial property of antisense *yycG* RNA on *Staphylococcus aureus*

**DOI:** 10.1186/s13018-019-1356-x

**Published:** 2019-09-06

**Authors:** Shizhou Wu, Yunjie Liu, Hui Zhang, Lei Lei

**Affiliations:** 1 Department of Orthopedics, West China Hospital, Sichuan University, No. 37 Guoxue Alley, Chengdu, 610041 Sichuan People’s Republic of China; 2 State Key Laboratory of Oral Diseases, Department of Preventive Dentistry, West China Hospital of Stomatology, Sichuan University, No. 14 Renmin South Road, Chengdu City, 610041 Sichuan China; 3 West China School of Public Health, Sichuan University, No. 18 Renmin South Road, Wuhou District, Chengdu City, China

**Keywords:** *Staphylococcus aureus*, Antisense RNA, YycG, Graphene oxide, Biofilm

## Abstract

**Background:**

*Staphylococcus aureus* (*S. aureus*) has the potential to opportunistically cause infectious diseases, including osteomyelitis, skin infections, pneumonia, and diarrhea. We previously reported that AS*yycG* RNA reduced the transcripts of virulent genes, and biofilm formation of *S. aureus*. Currently, graphene oxide (GO) nanosheets are used to efficiently deliver nucleic acids with favorable biocompatibility.

**Methods:**

In the current study, a GO-based recombinant pDL278 AS*yycG* vector transformation strategy was developed. The particle size distributions and zeta-potential of the GO-PEI-based AS*yycG* were evaluated. The AS*yycG* plasmids were labeled with gene-encoding enhanced green fluorescent protein (AS*yycG*-eGFP). Quantitative real-time PCR assays were performed to investigate the expression of *yycF/G/H* and *icaADB* genes. Biofilm biomass and bacterial viability of *S. aureus* were evaluated by scanning electron microscopy and confocal laser scanning microscopy. We found that the expression of the *yycG* gene was inversely correlated with levels of the AS*yycG* transcripts and that the GO-PEI-AS*yycG* strain had the lowest expression of biofilm organization-associated genes.

**Results:**

The results showed that the GO-based strategy significantly increased AS*yycG* transformation as a delivery system compared to the conventional competence-stimulating peptide strategy. Furthermore, GO-PEI-AS*yycG* suppressed bacterial biofilm aggregation and improved bactericidal effects on *S. aureus* after 24 h biofilm establishment.

**Conclusions:**

Our findings demonstrated that nano-GO with antisense *yycG* RNA is a more effective and relatively stable strategy for the management of *S. aureus* infections.

**Electronic supplementary material:**

The online version of this article (10.1186/s13018-019-1356-x) contains supplementary material, which is available to authorized users.

## Introduction

*Staphylococcus aureus* (*S. aureus*), a gram-positive coccus, is carried by about 20–30% of healthy individuals and mostly colonizes the nasopharynx [1]. *S. aureus* has the potential to cause a wide range of diseases, including osteomyelitis, skin infections, pneumonia, and even life-threatening infective endocarditis associated with considerable global human morbidity and mortality [2]. However, in some cases, *S. aureus* is resistant to multiple types of antibiotics, which has been attributed to the abuse of antibiotics, resulting in the emergence of methicillin-resistant *Staphylococcus aureus* (MRSA) [3]. Presently, more than 50% of *S. aureus* in clinical isolates in hospitals worldwide are methicillin-resistant [4]. Therefore, the identification of novel antibacterial strategies is of the utmost importance.

Graphene oxide (GO) is a graphene sheet containing functional organic groups, such as carboxyl, hydroxyl, carbonyl, and epoxy, on its basal plane [5]. The sharp edges on the GO sheet structures physically disrupt cell membranes and cause oxidative stress reactions. Therefore, it is expected to act as a novel type of bactericidal agent with a low risk of developing resistance from pathogenic bacteria [6]. In addition, the large surface area of GO sheets makes them ideal candidates for gene delivery [7]. Although GO sheets can be used to effectively deliver single-stranded nucleic acids, the ability of GO to carry double-stranded DNA (dsDNA) is limited. Polyethyleneimine (PEI) is a well-studied cationic polymer that has been used as a common non-viral gene delivery vector when combined with GO. Compared with the PEI polymer, GO–PEI has been reported to have lower cytotoxicity and higher transfection efficiency, and thus, has high potential as a gene vector [8].

Two-component signal transduction systems (TCSs) are essential pathways for bacterial responses to environmental stimuli. Typical TCS components consist of a transmembrane histidine kinase sensor and a corresponding cytoplasmic response regulator, which can bind to specific regions to regulate the expression of targeted genes [9]. YycFG is the only essential TCS in *S. aureus*, contributing to its physiology, and biofilm metabolism [10]. Biofilms are microbial communities embedded within self-produced extracellular substances and are closely related to the development of infections in humans [11]. In *S. aureus*, polysaccharide intercellular adhesion (PIA) encoded by *icaADBC* is a functional factor involved in biofilm organization [12].

Antisense RNAs (asRNAs) are a type of single-strand RNA that recognize mRNA by base-pairing and inhibiting the transcription and transduction of target mRNA [13]. Antisense RNA strategy is a promising approach for novel gene-specific antisense antibiotics to cure bacterial infections [14]. However, the efficiency of transforming antisense RNA into bacterial cells is limited without a suitable carrier system [15]. Since GO-PEI complexes are highly positively charged, effective loading with DNA plasmids can be achieved. In this study, a GO-based plasmid transformation system was developed using GO-PEI complexes that were loaded with antisense *yycG* plasmid (GO-PEI-AS*yycG*). We hypothesized that the antibacterial properties of GO to *S. aureus* could be enhanced by loading the gene vector with antisense AS*yycG* plasmids. A potential role for the clinical application GO-PEI-AS*yycG* as a novel antibiotic agent was proposed for the management of the *S. aureus* infections.

## Methods and materials

### Preparation of GO-PEI-ASyycG and cytotoxicity evaluation

The antisense *yycG* sequences (AS*yycG*) were synthesized by Sangon Biotech (Shanghai, China). To generate a recombinant pDL278 AS*yycG* plasmid, the AS*yycG* sequences were inserted into the BamHI and EcoRI restriction sites of a pDL278 vector [16]. To synthesize the GO-PEI complexes, GO powder (XFNANO Materials Tech, Nanjing, China) was added to ddH_2_O to a final concentration of 0.1 mg/mL. Next, the solution was slowly mixed with branched polyethyleneimine (BPEI, 10 kDa; Sigma-Aldrich, St. Louis, MO, USA). Then, the solution was processed with 10 cycles of ultrasonication for 60 s, with 60 s rest on ice between each sonication. The obtained solution was mixed on a shaking table overnight at room temperature. To remove redundant PEI compounds, the mixtures were washed three times with ddH_2_O by centrifugation (12000×*g*, 1 min) and resuspended with ddH_2_O to a final concentration of 0.1 mg/mL. pDL278 AS*yycG* plasmid (100ng/μL) was added to the GO-PEI complexes at a volume ratio of 1:125 and the mixtures were incubated for 1 h at room temperature.

The working concentration of GO-PEI-AS*yycG* was determined by cytotoxicity assays. Briefly, the mouse embryonic fibroblast NIH/3T3 cell line (Sigma-Aldrich) at a density of 1000 cells/well were seeded into 96-well plates in 100 μL of Dulbecco’s modified Eagle’s medium (DMEM), supplemented with 10% fetal bovine serum (FBS), and mixed with the GO-PEI-AS*yycG* solutions at dilutions ranging from 100 μg/mL to 0 μg/mL. After incubation for 48 or 72 h (37 °C, 5% CO_2_), the plates were removed and each well was washed with phosphate buffer solution (PBS, pH = 7.4) twice. The CCK-8 cell counting kit (Dojindo Laboratories, Kumamoto, Japan) was used to test the cell viability and each well was incubated with 10 μL of CCK-8 reagent. After 2 h of culture, the OD values of each well were measured using a microplate reader (ELX800, Gene, Hong Kong, China) at 540 nm.

### Particle size distribution, zeta potential, and atomic force microscopy measurements

The particle size distribution of the GO, GO-PEI, and GO-PEI-AS*yycG* solutions (0.1 mg/mL) was measured by dynamic light scattering (DLS) and the zeta-potential was examined by a Malvern instrument (Zetasizer Malvern Nano ZS, Instruments, Worcestershire, UK). A total of 50 μL of GO, GO-PEI, or GO-PEI-AS*yycG* solution was dropped onto sterile coverslips and films were prepared and air-dried in room temperature. The roughness of the films was assessed using an atomic force microscope (AFM) (SPM-9500J2, Shimadzu, Tokyo, Japan) in the contact mode. Micrographs of all films were evaluated by scanning electron microscopy (SEM; Inspect F50, FEI, Hillsboro, OR, USA) as previously described [17].

### Bacterial culture and transformation

A single colony of *S. aureus* was selected from a tryptic soy agar (TSA) plate and cultured in tryptic soy broth (TSB) medium to the mid-exponential phase, which was determined by an OD_600_ value of 0.5. For the *S. aureus* GO group, 250 μL of mid-exponential *S. aureus* was incubated with 2 μL GO solution (final concentration determined by cell viability assay). In the AS*yycG* group, 2 μL of recombinant pDL278 AS*yycG* plasmid was mixed with 250 μL of mid-exponential *S. aureus* as in our previous studies [16]. For the GO-PEI-AS*yycG* strains, 250 μL of mid-exponential *S. aureus* was co-cultured with prepared GO-PEI-AS*yycG*. All *S. aureus* strains were cultured at 37 °C in 5% CO_2_ for 1 hour, then diluted into 5 mL of fresh TSB medium.

### Transformation efficiency of GO-PEI-ASyycG in vitro

The AS*yycG* plasmids were labeled with gene encoding enhanced green fluorescent protein (AS*yycG*-eGFP). The sequences of AS*yycG* and eGFP were synthesized by Sangon Biotech (Shanghai, China) and are listed in the Additional file 1. The ASy*ycG*-eGFP and GO-PEI-AS*yycG*-eGFP strains were constructed based on the transformation procedures described above. Both strains were grown in TSB medium until an OD_600_ value of 0.5 was reached. A total of 50 μL of bacterial suspensions was dropped onto coverslips and air-dried at room temperature for 30 min. Confocal laser scanning microscopy (CLSM) was applied to determine the expression level of eGFP. The transfection efficiency was determined by comparing the green fluorescence intensities.

Real-time polymerase chain reaction (RT-PCR) assays were conducted to assess the expression of AS*yycG* in all *S. aureus* strains. Briefly, total RNA was extracted from *S. aureus* suspensions from the mid-logarithmic growth phase in TSB medium using an RNA purification Kit (MasterPure, Epicentre, Madison, WI, USA) according to the manufacturer’s instructions. Total RNA was reverse transcribed using an RT Reagent Kit (PrimeScript, Takara, Kyoto, Japan). Quantitative RT-PCR assays were carried out using the primers listed in Table 1 using a LightCycler 480 system (Roche, Basel, Switzerland). The 16S rRNA gene was used as an internal control [17].

**Table 1:** Sequences of primers used for qRT-PCR analysis

Primers	Sequence 5′-3′ (forward/reverse)	Reference
RT-qPCR		
*icaA*	5′-GATTATGTAATGTGCTTGGA-3′/ 5′-ACTACTGCTGCGTTAATAAT-3′	This study
*icaD*	5′-ATGGTCAAGCCCAGACAGAG-3′/ 5′-CGTGTTTTCAACATTTAATGCAA-3′	This study
*icaB*	5′-CACATACCCACGATTTGCAT-3′/ 5′-TCGGAGTGACTGCTTTTTCC-3′	This study
*yycF*	5′-TGGCGAAAGAAGACATCA-3′/ 5′-AACCCGTTACAAATCCTG-3′	This study
*yycG*	5′-CGGGGCGTTCAAAAGACTTT-3′/ 5′-TCTGAACCTTTGAACACACGT-3′	This study
*16S rRNA*	5′-GTAGGTGGCAAGCGTTATCC-3′/ 5′-CGCACATCAGCGTCAACA-3′	This study

### Growth conditions of S. aureus strains

After transformation, all strains were diluted in TSB at a ratio 1:20 and incubated in 96-well plates. The bacterial growth curves were monitored by measuring the OD_600nm_ with a microplate reader (ELX800, Gene, Hong Kong, China) every 60 min for 24 h. The proportions of live bacteria cells were estimated by confocal laser scanning microscopy (CLSM, FV1000; Olympus Corporation, Tokyo, Japan) at × 40 magnification. Live cells were stained with SYTO9 dye (LIVE/DEAD Bacterial Viability Kit reagent; BacLight, Invitrogen, Grand Island, NY, USA) and dead cells were labeled with propidium iodide (PI). Three-dimensional reconstruction was conducted and analyzed using Imaris 7.0.0 software (Imaris 7.0.0, Bitplane, Zurich, Switzerland) as previously described [18].

### Evaluation of S. aureus biofilms

Crystal violet (CV) assays were applied to compare the biomass of *S. aureus* biofilms cultured in 24-well polystyrene plates for 24 h. As previously described, the biofilms were stained with 0.1% (w/v) crystal violet for 15 min at room temperature [17]. The dye bound on the biofilms was collected using 1 mL of de-staining solution (8:2 ethanol: acetone). Then, the solution was transferred to a new plate and the OD_600nm_ was read by a microplate reader (ELX800, Gene, Hong Kong, China).

Sterile coverslips were immersed in 24-well plates with different *S. aureus* strain suspensions (OD_600nm_ = 0.5). After 24 h of co-culturing, the planktonic suspensions were removed and the biofilms grown on the coverslips were washed three times with PBS (pH7.2). Then, the biofilms were fixed in 2.5% glutaraldehyde for 4 h at room temperature and dehydrated with serially concentrated ethanol solutions (30%, 50%, 70%, 80%, 95%, and 100%). The prepared biofilms were dried to critical-point at room temperature and coated with gold powder. Scanning electron microscopy (SEM; Inspect F50, FEI, Hillsboro, OR, USA) was used to estimate the morphologies of all *S. aureus* biofilms by selecting three random areas from each sample [19].

### Data analyses

All data were processed using SPSS software (SPSS version 20, IBM, Armonk, NY, USA). The values are expressed as mean ± standard deviation (SD) for the indicated number of samples. The quantification cycles for describing gene expression were relatively quantified by real-time PCR using 16S as an internal control and calculated based on the ATCC29213 expression, which was set to 1.0. Bartlett’s test was employed to assess the homogeneity of data variance and the Shapiro-Wilk test was conducted to determine the normal distribution of the data. One-way analysis of variance was used to compare the data, followed by pairwise multiple comparisons. The differences were considered significant if the *p* value was < 0.05.

## Results

### Cytotoxicity and characteristics of GO-PEI-ASyycG films

**Fig. 1:**
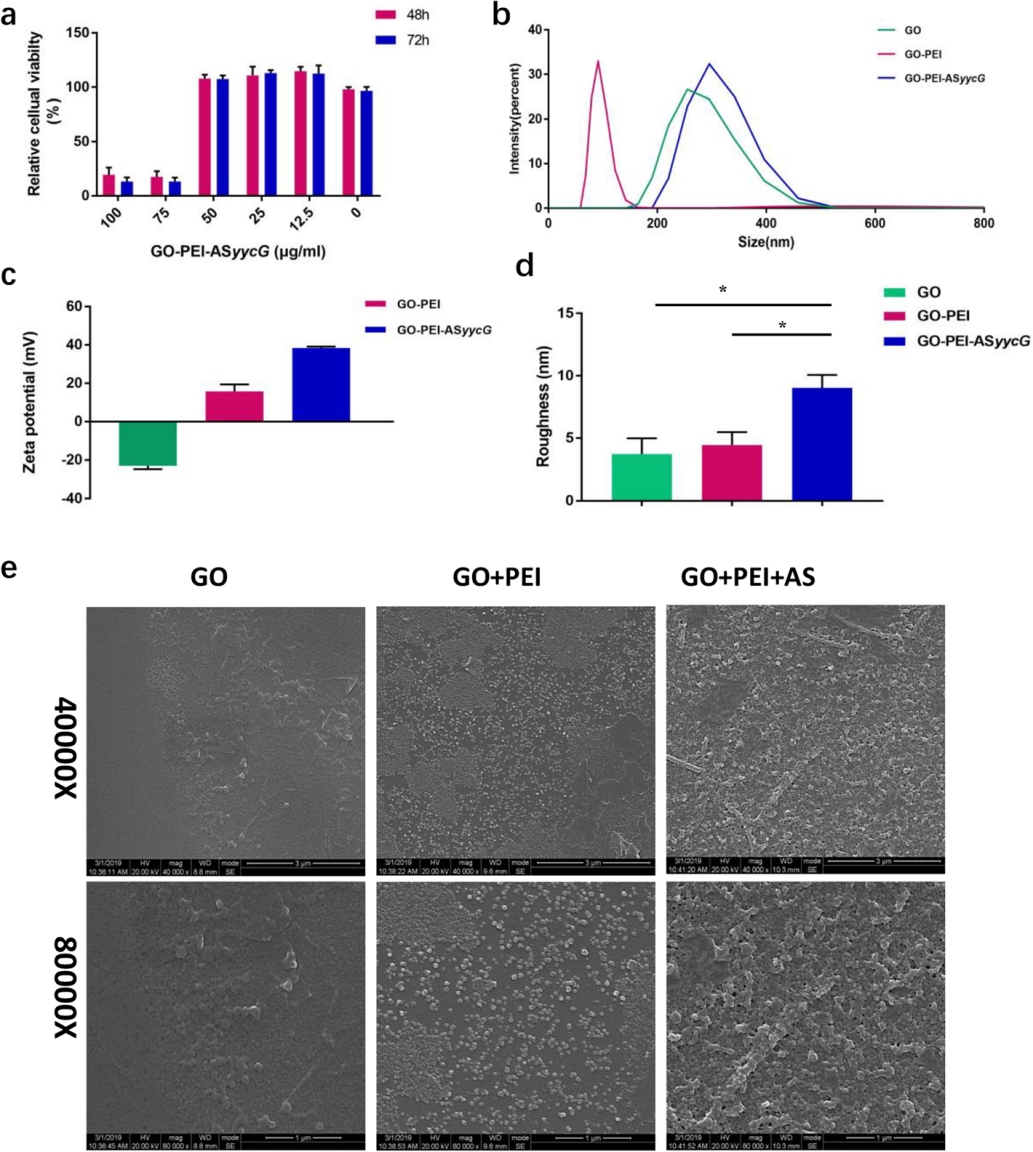
Cytotoxicity and characterization of GO-PEI-*yycG*. **a** The cell viability were determined with CCK-8 after 48 h or 72 h of incubation with GO-PEI-AS*yycG* (Dulbecco’s modified Eagle’s medium supplemented with 10% fetal bovine serum was the blank control). **b** The particle size distributions were measured using dynamic light scattering. **c** The zeta potential of the GO-PEI-based AS*yycG* was assessed by a Malvern Zetasizer. **d** AFM confirmed the roughness parameters of GO, GO-PEI, and GO-PEI-AS*yycG* material films (*n* = 10,*p < 0.05). **e** SEM images of GO, GO-PEI, and GO-PEI-AS*yycG* material films

The viability of the 3T3 fibroblasts cells was significantly decreased after 48 or 72 h treatment GO-PEI-AS*yycG* concentrations higher than 50 μg/mL (Fig. 1a). Our results indicated that the GO-PEI-AS*yycG* complexes were not toxic at concentrations lower than 50 μg/mL (Fig. 1a). Dynamic light scattering measurements were applied to determine the hydrodynamic sizes of GO, GO-PEI, and GO-PEI-AS*yycG* in deionized water. Z-average sizes of 420 nm and 280 nm were obtained for GO and GO-PEI, which were much smaller than the average size of GO-PEI-AS*yycG* (490 nm) (Fig. 1b). The surface charge (zeta potential) value of GO was approximately at − 23.1 mV. After mixing with the cationic PEI polymer, the GO-PEI and GO-PEI-AS*yycG* complexes demonstrated positive surface charges of 15.7 mV and 38.4 mV, respectively (Fig. 1c). Using AFM, the roughness analysis of the membranes revealed that the GO-PEI-AS*yycG* nanosheet roughness averaged 8.9 nm, significantly higher than the GO and GO-PEI films, which averaged 3.4 nm and 4.5 nm, respectively (*n* = 10, *p* < 0.05; Fig. 1d). Using SEM, the results showed rougher and denser surface morphologies on the GO-PEI-AS*yycG* films compared to GO and GO-PEI (Fig. 1e).

### GO-PEI-ASyycG increased ASyycG transformation and significantly reduced biofilm formation-associated gene expression

**Fig. 2:**
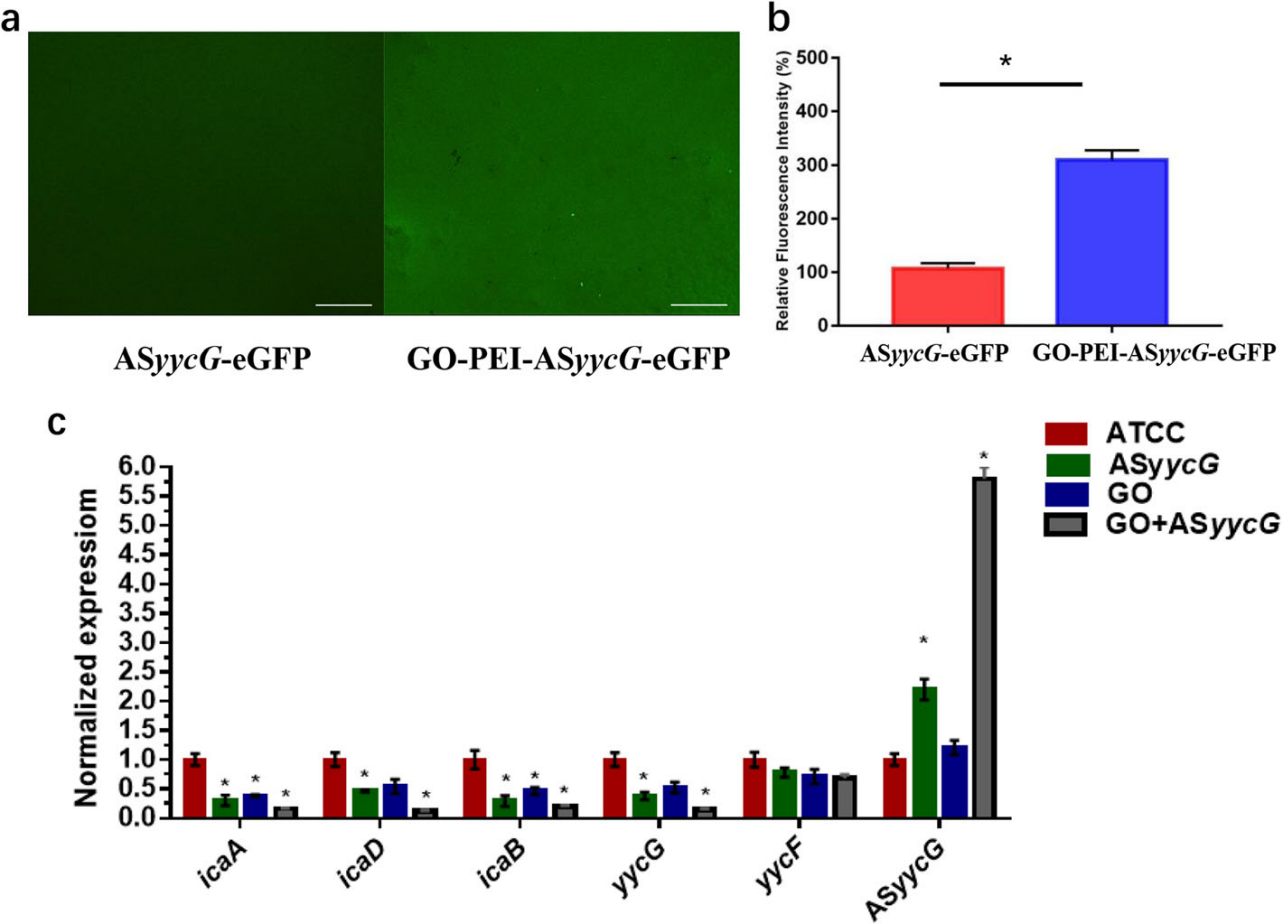
GO-PEI-AS*yycG* increased AS*yycG* transformation and inhibited virulence gene expressions. **a** AS*yycG* plasmids were labeled with gene encoding enhanced green fluorescent protein (AS*yycG*-eGFP) and CLSM was applied to determine the expression level of eGFP. **b** The transfection efficiency was determined by comparing the green fluorescent intensities (*n* = 10, *p < 0.05). **c** Quantitative RT-PCR analysis showed gene transcription in untreated *S. aureus* and AS*yycG*-, GO-, and GO-PEI-AS*yycG*-treated strains; *S. aureus* gene expression was quantified relatively using *16sR* as an internal control and calculated based on untreated *S. aureus* ATCC29213 expression, which was set as 1.0. (*n* = 10, *p < 0.05)

Using confocal laser scanning microscopy, higher levels of GFP-expression were observed in samples induced with GO-PEI-AS*yycG* compared to pure AS*yycG* (Fig. 2a). A 200% increase in GFP-expression transcripts in GO-PEI-AS*yycG* transformed strains was found, a statistically significant increase compared to the expression in AS*yycG* cells (*n* = 10, *p* < 0.05; Fig. 2b). Quantitative RT-PCR assays demonstrated that expression levels of AS*yycG* RNA in the AS*yycG* and GO-PEI-AS*yycG* strains were significantly increased 2.8-fold and 6.5-fold, respectively, compared to *S. aureus* ATCC29213 strains (*n* = 10, *p* < 0.05; Fig. 2c). Accordingly, the gene expression levels of *yycG* were significantly downregulated in the AS*yycG* and GO-PEI-AS*yycG* strains (*n* = 10, *p* < 0.05). Consequently, the expression levels of PIA synthesis-associated genes *icaA/D/B/C* were the lowest in the GO-PEI-AS*yycG* strain among all groups (*n* = 10, *p* < 0.05).

### GO-PEI-ASyycG decreased cells viability and suppressed biofilm formation

**Fig. 3:**
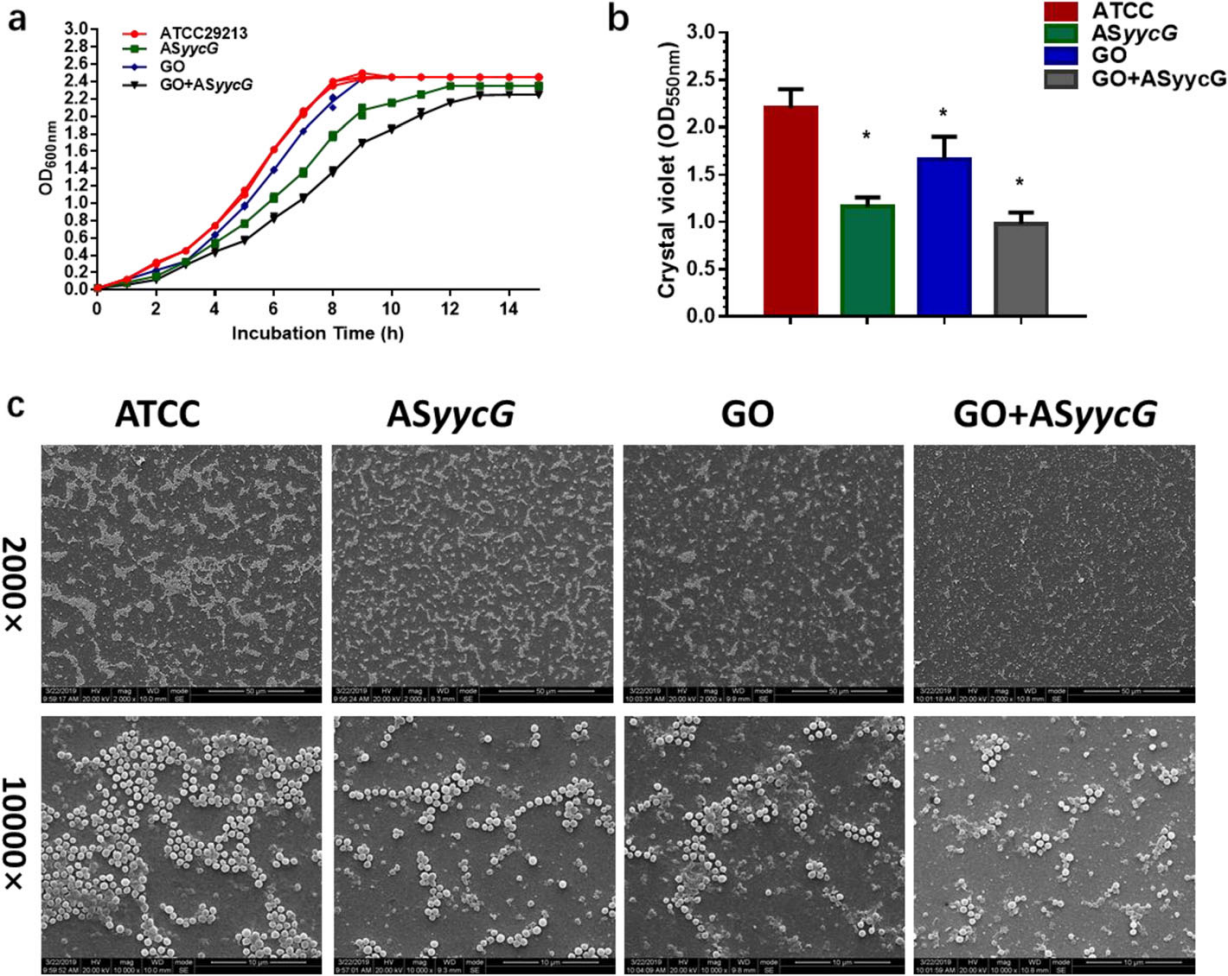
GO-PEI-AS*yycG* suppressed *S. aureus* growth and biofilm formation. **a** *S. aureus* and AS*yycG*-, GO-, and GO-PEI-AS*yycG*-treated strains were cultured and growth was monitored every hour. **b** Biomass was quantified by crystal violet staining. Optical densities at 600 nm were measured (*n* = 10, *p < 0.05). **c** SEM of untreated *S. aureus* and AS*yycG-*, GO-, and GO-PEI-AS*yycG*-treated strains for 24 h biofilms. Scale bar for × 2000 magnification, 50 μm. Scale bar for × 10,000 magnification, 10 μm

When growth was monitored in the different strains, the results showed that the time before entry into the log phase in the GO-PEI-AS*yycG* strains was obviously prolonged compared to that of the AS*yycG* strains (Fig. 3a). The most impaired formation of biofilms was observed in GO-PEI-AS*yycG* strains compared to GO, AS*yycG*, and ATCC29213 (Fig. 3b). Quantitatively, these results were confirmed by the OD values of the biofilm biomasses, which were the lowest in the GO-PEI-AS*yycG* strains (*n* = 10, *p* < 0.05, Fig. 3b).

**Fig. 4:**
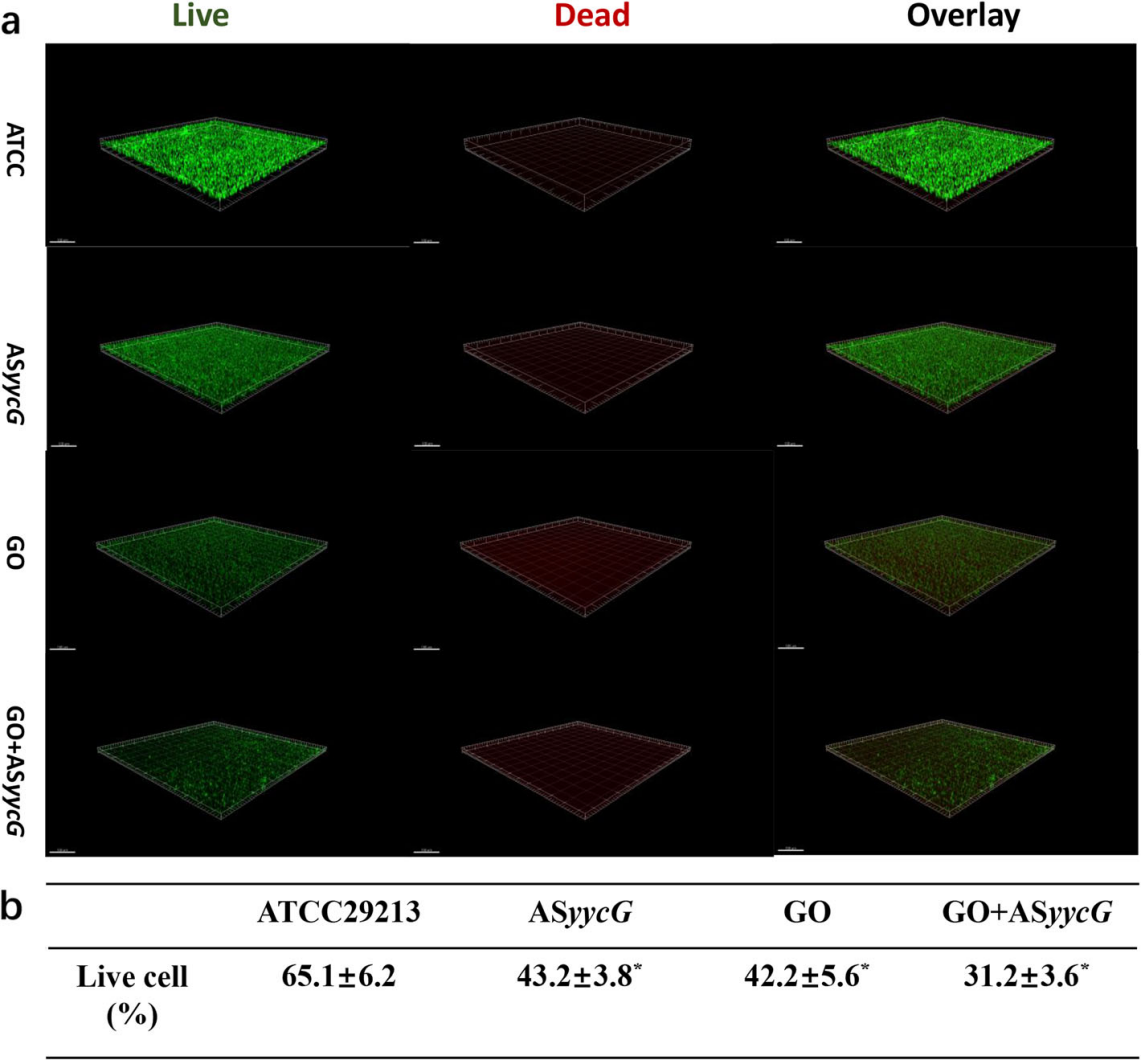
GO-PEI-AS*yycG* suppressed the vital cells in *S. aureus* biofilms. **a** Double labeling of the biofilms in the untreated *S. aureus* and AS*yycG-*, GO-, and GO-PEI-AS*yycG*-treated strains. Green, vital cells (SYTO 9); red, dead cells (PI); scale bars, 100 μm. The three-dimensional reconstruction of the biofilms was performed using Imaris 7.0.0. **b** Volume ratio of the vital bacterial biomass in the biofilms (*n* = 10, *p < 0.05)

SEM observation demonstrated reduced levels of extracellular matrix components in the biofilms of the GO, AS*yycG*, and GO-PEI-AS*yycG* cells that were separated by blank areas (Fig. 3c). Exopolysaccharide-enmeshed cell clusters were greatly decreased in the GO-PEI-AS*yycG* strain biofilms (Fig. 3c). Using CLSM, we demonstrated that the proportion of viable bacteria was significantly decreased in the GO, AS*yycG*, and GO-PEI-AS*yycG* strains compared to the *S. aureus* ATCC29213 strain (Fig. 4a). The lowest percentage of live bacteria was observed in the GO-PEI-AS*yycG* strain, at 14.83 ± 0.5 % (*n* = 10, *p* < 0.05, Fig. 4b).

## Discussion

Our previous work indicated that antisense *yycG* RNA (AS*yycG*) could inhibit the target gene *yycG* in the MRSA strain. We found that AS*yycG* strains inhibited biofilm organization and increased antibiotic sensitivity [20]. However, one of the major obstacles to the use of antisense oligonucleotides is that, without a suitable and effective vector, the uptake by bacterial cells is limited [21]. In the current study, a GO-based recombinant pDL278 AS*yycG* vector transformation strategy was used to electrostatically combine the vector with cationic GO-PEI complexes. We showed that GO-PEI could efficiently deliver AS*yycG* plasmid into *S. aureus* cells with efficient transcripts of AS*yycG*. GO has been reported to ionically bind to cationic PEI polymers [8]. These positive surface charges could interact with the negatively charged cellular surface and promote bacterial transformation [22]. A previous study reported that 50 μg/mL of GO-PEI (or lower) did not have toxic effects on the cellular apoptosis rate [23]. In the present study, we demonstrated that the synthesized GO-PEI-AS*yycG* was not toxic at concentrations less than 50 μg/mL (Fig. 1a). Therefore, 50 μg/mL of GO-PEI-AS*yycG* was adopted as the working concentration.

The results of AFM observation indicated that the surface roughness of the GO-PEI-AS*yycG* nanosheets was increased compared to GO and GO-PEI material films (Fig. 1d). The surface characteristics of the GO nanosheets films were assessed using SEM, which showed a rougher and denser surface morphology in the GO-PEI-AS*yycG* films compared to those of GO and GO-PEI (Fig. 1e). Because the surface roughness of membrane films could influence bacterial colonization and adhesion, the increased surface characteristics of the GO-PEI-AS*yycG* material films probably indicated enhanced adhesive force.

To evaluate the vector transformation efficiencies, AS*yycG* recombinant plasmids were labeled with gene encoding enhanced green fluorescent protein. The levels of GFP-expression indicated the presence of AS*yycG* transcripts and revealed higher transformation efficiencies in *S. aureus* cells induced by GO-PEI-AS*yycG* compared to pure AS*yycG* plasmids. In particular, the quantitative RT-PCR assays showed that the fold change in AS*yycG* expression in the GO-PEI-AS*yycG* strain was roughly 3-fold higher in the AS*yycG* strain transformed with competence-stimulating peptide. We speculated that the GO-based strategy significantly increased AS*yycG* transformation as a delivery system and reduced transcripts of the *yycG* gene.

Furthermore, GO-PEI-AS*yycG* significantly suppressed bacterial growth and biofilm aggregation (Fig. 3). Using SEM observation, few randomly distributed microcolonies were identified and exopolysaccharide-enmeshed cell clusters were greatly decreased in the GO-PEI-AS*yycG* strain biofilms compared to the GO and AS*yycG* strains. After 24 h of biofilm establishment, CLSM findings revealed that GO-PEI-AS*yycG* greatly reduced the cellular viability (Fig. 4). These results suggested that *S. aureus* was markedly inhibited by GO-PEI-AS*yycG*, which improved the bactericidal effects of AS*yycG* on the *S. aureus* biofilms.

Biofilm-forming capacity is an essential factor in the development of *S. aureus*-induced infections and results in significant increases in morbidity and mortality [24]. In *S. aureus*, PIA is a crucial component for biofilm organization [25], which is mostly synthesized by glycosyltransferase enzymes encoded by the *ica* operon [12, 26]. In the current study, the GO-PEI-AS*yycG* strain had the lowest expression of *yycF/G/H* and *icaA/D* genes and biofilm formation, indicating that the pathogenesis of *S. aureus* was further decreased by the GO-PEI complexes, improving AS*yycG* transformation. Injectable GO-PEI-AS*yycG* could be useful in orthopedic applications to manage osteomyelitis lesions and reduce the use of antibiotics. Future directions will need to extend the applications of GO-PEI-AS*yycG* strategy as a potential way of managing the antibiotic resistance of *S. aureus* infections. At an appropriate concentration, GO-PEI-AS*yycG* could potentially improve the antibacterial properties of irrigation fluid. However, a limitation of the current study was the lack of in vivo experiments which are needed to confirm the effective concentration of this novel antibacterial agent before clinical application.

In the current study, a GO-based recombinant pDL278 AS*yycG* vector transformation strategy was developed. We found that the expression of the *yycG* gene was inversely correlated with the levels of AS*yycG* transcripts and that the GO-PEI-AS*yycG* strain had the lowest expression of biofilm organization-associated genes. The GO-based strategy significantly increased AS*yycG* transformation as a delivery system compared to the conventional competence-stimulating peptide strategy. Furthermore, GO-PEI-AS*yycG* suppressed aggregation of bacterial biofilms and improved the bactericidal effects on *S. aureus* after 24 h of biofilm establishment. Thus, our data demonstrated that nano-GO with antisense *yycG* RNA may be an effective and relatively stable strategy for the management of *S. aureus* infections.

## Additional files

Additional file 1: The sequences of ASyycG and eGFP were synthesized by Sangon Biotech (Shanghai, China) and were inserted into BamHI and EcoRI restriction sites of a pDL278 vector. The initial sites of eGFP were underlined and bold in red. (DOCX 14 kb)

## Data Availability

All data generated or analyzed during this study are included in this published article and its supplementary information files.

## Abbreviations

AFM: Atomic force microscopy
CLSM: Confocal laser scanning microscope
CV: Crystal violet
EPS: Extracellular polymeric substances
GO: Graphene oxide
PIA: Polysaccharide intercellular adhesion
*S. aureus*: *Staphylococcus aureus*
SEM: Scanning electron microscopy

## References

[1] van Belkum A, Verkaik NJ, de Vogel CP, Boelens HA, Verveer J, Nouwen JL, Verbrugh HA, Wertheim HF (2009). Reclassification of Staphylococcus aureus nasal carriage types. J Infect Dis..

[2] Boada A, Pons-Vigués M, Real J, Grezner E, Bolíbar B, Llor C (2018). Previous antibiotic exposure and antibiotic resistance of commensal Staphylococcus aureus in Spanish primary care. Eur J Gen Pract..

[3] Harkins CP, Pichon B, Doumith M, Parkhill J, Westh H, Tomasz A, de Lencastre H, Bentley SD, Kearns AM, Holden MTG (2017). Methicillin-resistant *Staphylococcus aureus* emerged long before the introduction of methicillin into clinical practice. Genome Biol..

[4] Stefani S, Chung DR, Lindsay JA, Friedrich AW, Kearns AM, Westh H, Mackenzie FM (2012). Meticillin-resistant *Staphylococcus aureus* (MRSA): global epidemiology and harmonisation of typing methods. Int J Antimicrob Agents..

[5] Zarafu I, Turcu I, Culiță DC, Petrescu S, Popa M, Chifiriuc MC, Limban C, Telehoiu A, Ioniță P (2018). Antimicrobial features of organic functionalized graphene-oxide with selected amines. Materials (Basel)..

[6] Liu Y, Yuan C, Cheng Y, Yao G, Xie L, Xu B (2018). Graphene oxide affects growth and resistance to Sclerotinia sclerotiorum in Brassica napus L. J Nanosci Nanotechnol..

[7] Di Santo R, Digiacomo L, Palchetti S, Palmieri V, Perini G, Pozzi D, Papi M, Caracciolo G (2019). Microfluidic manufacturing of surface-functionalized graphene oxide nanoflakes for gene delivery. Nanoscale..

[8] Feng L, Zhang S, Liu Z (2011). Graphene based gene transfection. Nanoscale..

[9] Fukuchi K, Kasahara Y, Asai K, Kobayashi K, Moriya S, Ogasawara N (2000). The essential two-component regulatory system encoded by *yycF* and *yycG* modulates expression of the ftsAZ operon in *Bacillus subtilis*. Microbiology..

[10] Dubrac S, Msadek T (2008). Tearing down the wall: peptidoglycan metabolism and the WalK/WalR (YycG/YycF) essential two-component system. Adv Exp Med Biol..

[11] Flemming HC, Wingender J, Szewzyk U, Steinberg P, Rice SA, Kjelleberg S (2016). Biofilms: an emergent form of bacterial life. Nat Rev Microbiol..

[12] O’Gara JP (2007). ica and beyond: biofilm mechanisms and regulation in Staphylococcus epidermidis and *Staphylococcus aureus*. FEMS Microbiol Lett..

[13] Saberi F, Kamali M, Najafi A, Yazdanparast A, Moghaddam MM (2016). Natural antisense RNAs as mRNA regulatory elements in bacteria: a review on function and applications. Cell Mol Biol Lett..

[14] Bai H, Xue X, Hou Z, Zhou Y, Meng J, Luo X (2010). Antisense antibiotics: a brief review of novel target discovery and delivery. Curr Drug Discov Technol..

[15] Zhang Y, Ma W, Zhu Y, Shi S, Li Q, Mao C, Zhao D, Zhan Y, Shi J, Li W, Wang L, Fan C, Lin Y (2018). Inhibiting methicillin-resistant *Staphylococcus aureus* by tetrahedral DNA nanostructure-enabled antisense peptide nucleic acid delivery. Nano Lett..

[16] Lei L, Stipp RN, Chen T, Wu SZ, Hu T, Duncan MJ (2018). Activity of Streptococcus mutans VicR Is Modulated by Antisense RNA. J Dent Res..

[17] Wu S, Huang F, Zhang H, Lei L (2019). *Staphylococcus aureus* biofilm organization modulated by YycFG two-component regulatory pathway. J Orthop Surg Res..

[18] Wu S, Liu Y, Zhang H, Lei L (2019). The susceptibility to calcium hydroxide modulated by the essential *walR* gene reveals the role for *Enterococcus faecalis* biofilm aggregation. J Endod..

[19] Lei L, Yang Y, Mao M, Li H, Li M, Yang Y, Yin J, Hu T (2015). Modulation of biofilm exopolysaccharides by the *Streptococcus mutans vicX* Gene. Front Microbiol..

[20] Wu Shizhou, Liu Yunjie, Lei Lei, Zhang Hui (2019). Antisense yycG Regulation of Antibiotic Sensitivity of Methicillin-Resistant Staphylococcus aureus in Chronic Osteomyelitis. Surgical Infections.

[21] Bessa LJ, Ferreira M, Gameiro P (2018). Evaluation of membrane fluidity of multidrug-resistant isolates of *Escherichia coli* and *Staphylococcus aureus* in presence and absence of antibiotics. J Photochem Photobiol B..

[22] Quijano E, Bahal R, Ricciardi A, Saltzman WM, Glazer PM (2017). Therapeutic peptide nucleic acids: principles, limitations, and opportunities. Yale J Biol Med..

[23] Dou C, Ding N, Luo F, Hou T, Cao Z, Bai Y, Liu C, Xu J, Dong S (2017). Graphene-based microRNA transfection blocks preosteoclast fusion to increase bone formation and vascularization. Adv Sci (Weinh)..

[24] Moormeier DE, Bayles KW (2017). *Staphylococcus aureus* biofilm: a complex developmental organism. Mol Microbiol..

[25] Archer NK, Mazaitis MJ, Costerton JW, Leid JG, Powers ME, Shirtliff ME (2011). *Staphylococcus aureus* biofilms: properties, regulation, and roles in human disease. Virulence..

[26] Jenkins A, Diep BA, Mai TT, Vo NH, Warrener P, Suzich J, Stover CK, Sellman BR (2015). Differential expression and roles of *Staphylococcus aureus* virulence determinants during colonization and disease. MBio..

