# Supplementary material for: Nano-graphene oxide improved the antibacterial property of antisense yycG RNA on Staphylococcus aureus
Source: J Orthop Surg Res. 2019 Sep 6;14:305. doi: 10.1186/s13018-019-1356-x (PMC6731568; doi:10.1186/s13018-019-1356-x)
Supplement: Supplementary file 1 — The sequences of ASyycG and eGFP were synthesized by Sangon Biotech (Shanghai, China) and were inserted into BamHI and EcoRI restriction sites of a pDL278 vector. The initial sites of eGFP were underlined and bold in red. (DOCX 14 kb) [file 13018_2019_1356_MOESM1_ESM.docx]

The sequences of ASyycG and eGFP were synthesized by Sangon Biotech (Shanghai, China) and were inserted into BamHI and EcoRI restriction sites of a pDL278 vector. The initial sites of eGFP were underlined and bold in red

**ggatcc**TACCATAAATCTTCACACGTTGCGTATAGTTACCTCTGGACATTTCGACCGTCTGGTTACGCATATCGGTGATTGGTTTGGTAATCGTTCGCGCTATAAAGAATCCTAGGATGACTGTGATTAATAATGAAATAGCTGTACCAACAATGAATATTTGATTTATATTATTTAATTGGTTATAAACGTCATTAATTTTTGATTCGATATAAATATTACCAATTACCTTTTTATCGACTTTAACTGGGATATTATATACCCAGACACGGTCCTTACCACCGCCATAATCTTTTAAAATTAAATGATCGTTTGATTGTCCTAGTGATAGTGCTTTTTGGACAGAACTATCATTCGCTTTTTGATTGATTAGACTACGGTTAGACTGCTTCGTCGTCGCAATAATAATTTGGTCTTTATCTATAAAACGAATTTCTCCAATTTCTTGACGGTTGGCATACTCACTTAATAAATTTTGAATATCTTTTTGTGCATTTACGGAGCCCTTTTCGTCATATACTTTTTCAATACTAATTTCTAATTGTTTAGCGTACTGCGTAATATTCTTCTTAAAATTATCAAGCAGCTCTTTTTCAAGGTTATTTGTAAAATACAGCCCGATAATTTGCATACCAATGATAATCAGTAATACATAAACAATTACAAGTTTAGTATGAAGGGATTGTAGTTGTTTTAGCCACTTCAT**ATG**GTGAGCAAGGGCGAGGAGCTGTTCACCGGGGTGGTGCCCATCCTGGTCGAGCTGGACGGCGACGTAAACGGCCACAAGTTCAGCGTGTCCGGCGAGGGCGAGGGCGATGCCACCTACGGCAAGCTGACCCTGAAGTTCATCTGCACCACCGGCAAGCTGCCCGTGCCCTGGCCCACCCTCGTGACCACCCTGACCTACGGCGTGCAGTGCTTCAGCCGCTACCCCGACCACATGAAGCAGCACGACTTCTTCAAGTCCGCCATGCCCGAAGGCTACGTCCAGGAGCGCACCATCTTCTTCAAGGACGACGGCAACTACAAGACCCGCGCCGAGGTGAAGTTCGAGGGCGACACCCTGGTGAACCGCATCGAGCTGAAGGGCATCGACTTCAAGGAGGACGGCAACATCCTGGGGCACAAGCTGGAGTACAACTACAACAGCCACAACGTCTATATCATGGCCGACAAGCAGAAGAACGGCATCAAGGTGAACTTCAAGATCCGCCACAACATCGAGGACGGCAGCGTGCAGCTCGCCGACCACTACCAGCAGAACACCCCCATCGGCGACGGCCCCGTGCTGCTGCCCGACAACCACTACCTGAGCACCCAGTCCGCCCTGAGCAAAGACCCCAACGAGAAGCGCGATCACATGGTCCTGCTGGAGTTCGTGACCGCCGCCGGGATCACTCTCGGCATGGACGAGCTGTACAAGTAA**gaattc**
